# Resilience of native ant community against invasion of exotic ants after anthropogenic disturbances of forest habitats

**DOI:** 10.1002/ece3.9073

**Published:** 2022-07-11

**Authors:** Hiroyuki Shimoji, Mayuko Suwabe, Tomonori Kikuchi, Hitoshi Ohnishi, Hirotaka Tanaka, Kengo Kawara, Yusuke Hidaka, Tsutomu Enoki, Kazuki Tsuji

**Affiliations:** ^1^ School of Biological and Environmental Sciences Kwansei Gakuin University Hyogo Japan; ^2^ Okinawa Environmental Research Support Section Okinawa Institute of Science and Technology Graduate University Okinawa Japan; ^3^ Biodiversity and Biocomplexity Unit Okinawa Institute of Science and Technology Graduate University Okinawa Japan; ^4^ Marine Biosystems Research Center Chiba University Chiba Japan; ^5^ Kanto Regional Environment Office Ministry of the Environment Government of Japan Saitama Japan; ^6^ Faculty of Agriculture Ehime University Ehime Japan; ^7^ The Kyushu University Museum Fukuoka Japan; ^8^ Faculty of Agriculture Kyusyu University Fukuoka Japan; ^9^ Faculty of Agriculture University of the Ryukyus Okinawa Japan

**Keywords:** ant community, dispersal strategy, disturbance, invasive species, life‐history traits, *Technomyrmex brunneus*

## Abstract

The positive association between disturbances and biological invasions is a widely observed ecological pattern in the Anthropocene. Such patterns have been hypothesized to be driven by the superior competitive ability of invaders or by modified environments, as well as by the interaction of these factors. An experimental study that tests these hypotheses is usually less feasible, especially in protected nature areas. An alternative approach is to focus on community resilience over time after the anthropogenic disturbance of habitats. Here, we focused on ant communities within a forest to examine their responses after disturbance over time. We selected the Yanbaru region of northern Okinawa Island, which is a biodiversity hotspot in East Asia. We compared ant communities among roadside environments in forests where the road age differed from 5 to 25 years. We also monitored the ant communities before and after disturbance from forest thinning. We found that the species richness and abundance of exotic ants were higher in recently disturbed environments (roadsides of 5–15 years old roads), where the physical environment was warmer and drier. In contrast, the roadsides of 25‐year‐old roads indicated the potential recovery of the physical environment with cooler and moister conditions, likely owing to regrowth of roadside vegetation. At these sites, there were few exotic ants, except for those immediately adjacent to the road. The population density of the invasive species *Technoymex brunneus* substantially increased 1–2 years after forest thinning. There was no evidence of the exclusion of native ants by exotic ants that were recorded after disturbance. Our results suggest that local ant communities in the Yanbaru forests have some resilience to disturbance. We suggest that restoration of environmental components is a better strategy for maintaining native ant communities, rather than removing exotic ants after anthropogenic disturbance.

## Graphical abstract

The positive association between disturbances and biological invasions is a widely observed ecological pattern in the Anthropocene. We focused on ant communities within a forest to examine their responses after disturbance over time. Our results suggest that restoration of environmental components is a better strategy for maintaining native ant communities, rather than removing exotic ants after anthropogenic disturbance.

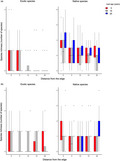

## 1 INTRODUCTION

The resilience of ecological communities is an important concept in the Anthropocene for biodiversity conservation. Invasion by exotic species is one of the main causes of biodiversity loss worldwide (Bellard et al., 2016; Vitousek et al., 1996; Wilcove et al., 1998). This is based on empirical data showing a negative relationship for species richness and abundance between exotic and native species. The exclusion of native species is caused by the superior competitive ability of exotic species, known as the “driver” hypothesis. This relates to ecological mechanisms, such as release from natural enemies (Mitchell & Power, 2003; Torchin et al., 2003; Wang et al., 2009), the use of empty niches that native species do not use (Elton, 1958; Shea & Chesson, 2002; Wang et al., 2009), and the possession of novel weapons against which native species have no resistance (Wang et al., 2009). However, some researchers have different views on biological invasions, suggesting that if exotic species have superior competitive abilities over native species, they ought to dominate any habitats that they can potentially inhabit (Didham et al., 2005; Gurevitch & Padilla, 2004; Hackerott et al., 2013). However, empirical evidence demonstrates that exotic species in introduced areas tend to show disturbance dependence. They usually become dominant in artificially modified environments, such as urban parks, roadsides, and agro‐ecosystems (Didham et al., 2005; Gurevitch & Padilla, 2004; Jauni et al., 2015). This has led to the idea that the invasion of an exotic species is not due to its superior competitive ability, but also the interaction between environmental disturbance and competition, known as the “back‐seat driver” hypothesis (Bauer, 2012). The removal of dominant native competitors through disturbance (Shea & Chesson, 2002) and/or the unstable equilibrium in which disturbance triggers the shift from a native ant‐dominated equilibrium to an exotic ant‐dominated equilibrium (Gause, 1935) can fall into this category. Some authors have suggested that the disturbance is the direct cause of the invasion, which is known as the “passenger” hypothesis (MacDougall & Turkington, 2005). Under this scenario, it can be hypothesized that disturbance modifies the physical environment, such as temperature and moisture, leading to suitable conditions for an invader to flourish.

There have been many discussions about the causal relationship between biological invasions and anthropogenic disturbance, and it is often difficult to empirically disentangle the driving factors (MacDougall & Turkington, 2005; Murray & Phillips, 2010; Salyer et al., 2014; Weir & Salice, 2012). However, the relative importance of competitive ability and environmental modification for invasion success is especially important for policy decisions regarding local biodiversity protection. If the back‐seat driver hypothesis is valid, conservation measures should focus on direct control of invasive species and restoration of environmental properties. If the passenger hypothesis is valid, it may be more important to restore the habitat than to control the invader population (Bauer, 2012). By physically restoring the habitat to its pre‐disturbance condition, the native community may also show resilience.

To examine this issue, we focused on ant communities. Ants are dominant in many terrestrial ecosystems and exotic ants can have a substantial impact on native biodiversity (Holway et al., 2002). A positive relationship between exotic species dominance and disturbance has also been established for ants (Arnan et al., 2018; Holway et al., 2002; Menke et al., 2018). Although competitive interaction is regarded as a hallmark of ant communities (Hölldobler & Wilson, 1990), alternative views on the disturbance‐driven establishment of invasive ants have also been explored (Bauer, 2012; Foucaud et al., 2010; King & Tschinkel, 2008; LeBrun et al., 2012; Roura‐Pascual et al., 2010; Stuhler & Orrock, 2016; Vonshak & Gordon, 2015). King and Tschinkel (2008) provided empirical evidence that artificial modification of the environment causes the settlement of the red imported fire ant, *Solenopsis invicta* (Stuble et al., 2011; Roeder et al., 2021). Dependent colony‐founding (fission and budding) is a characteristic of many invasive ants and is considered an important factor for rapid expansion in disturbed areas (Blumenfeld et al., 2021; Nakamaru et al., 2007, 2014; Tsuji & Tsuji, 1996). It is often difficult to decouple factors involved in disturbance and invasion in observation‐based empirical studies. Therefore, an experimental approach, such as habitat disturbance is also needed (Goodman & Warren II, 2019; King & Tschinkel, 2008, 2013; MacDougall & Turkington, 2005).

We used a semi‐experimental approach that focused on anthropogenic disturbance caused by governmental activities, rather than those planned by the researchers. We selected the Yanbaru region of northern Okinawa Island, Japan, as the study site. This area was designated as a national park in 2015 and as a Natural World Heritage Site in 2021. Yanbaru is of considerable interest in the study of community resilience. Although there are extensive forests with large areas of wilderness, most of these have been subjected to logging or other artificial modifications in the past (Abe et al., 2021). A large number of spectacular endemic animals, such as the Okinawa Rail (*Gallirallus okinawae*) and Okinawa Woodpecker (*Dendrocopos noguchii*) still inhabit the area, with no faunal species extinctions reported to date. This may suggest community resilience to human modification of the environment. Multiple exotic ants have already invaded the region (Suwabe et al., 2009; Tanaka et al., 2011; Yamauchi & Ogata, 1995), including *Pheidole megacephala* and *Anoplolepis gracilipes*, which are rated by the International Union for the Conservation of Nature (IUCN) as being among the 100 most invasive species worldwide. Yamauchi and Ogata (1995) reported that in Yanbaru, exotic ants tend to be rare in old forest stands where large trees are present with breast height diameters that exceed 50 cm, in comparison with younger forests and open lands. In old forest stands in Yanbaru, exotic ants are common in roadside environments along paved roads (Suwabe et al., 2009; Tanaka et al., 2011). This suggests that road construction may negatively impact the biodiversity of native ants. Therefore, it is important to investigate whether native ant communities will show resilience to this environmental modification through road construction.

We compared species richness and abundance for exotic and native ants among roadside environments in forested areas in Yanbaru, where the time since road construction varied from approximately 5–25 years. We focused on whether invasion by exotic ants would continue to expand in roadside environments following road construction and whether native ant communities in roadside environments would show resilience over time. We monitored changes in ant communities in the same plot before and after forest thinning over 7 years to examine whether changes in the ant communities could be directly observed.

## 2 MATERIALS AND METHODS

### 2.1 Roadside transect study

**FIGURE 1:**
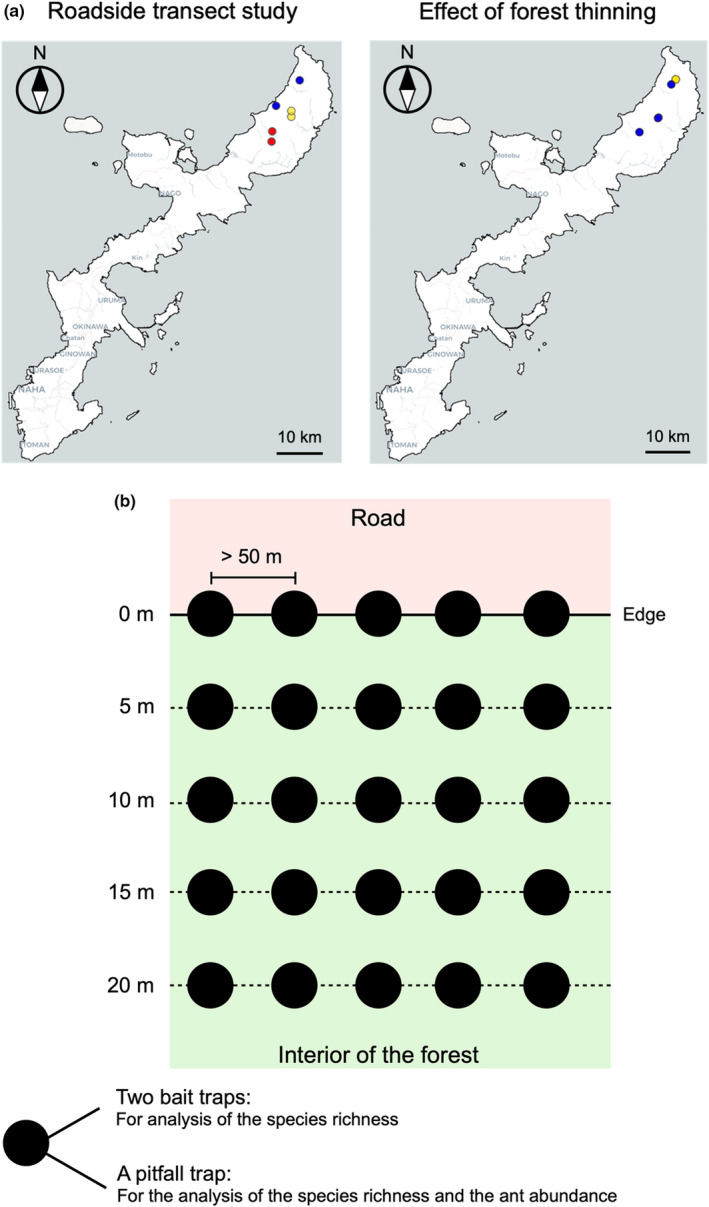
Experimental sites and procedures of the field survey (the Yanbaru region in the northern part of Okinawa Island, Japan). (a) Study sites of the roadside transect study. Blue, red, and yellow circles indicate 5, 15, and 25 years since road construction, respectively (left panel). Study sites for examining the effects of forest thinning on the ant community. Yellow and blue circles indicate thinned and control sites, respectively (right panel). (b) The setting design of the line transects in a site of the roadside transect study. Black circles indicate the positions of the traps (survey points). In this survey, we focused on three value types to evaluate the ant community. We used the number of ant individuals collected using a pitfall trap at each specific survey point as the record of ant abundance. The total number of ant species collected using two bait pitfall traps and a pitfall trap at a survey point was used as the species richness

The field survey for the roadside transect study was undertaken twice in a forested area in the northern part of Okinawa Island (Yanbaru). The first field survey was conducted in June 2005 and the second field survey was undertaken in October 2006 (Figure 1a). We were permitted to study the surrounding environments of six paved forest roads that had been constructed in different years (two each that were 5, 15, and 25 years old) as study sites (Figure 1 and Table S1). The environment along these roads was forest stands that had not been widely cut for more than 50 years. Note that there were no other suitable study sites other than those surveyed. Forest stands older than 50 years are rare and the management of the areas selected was under the control of the local government. However, the backgrounds of these six sites may not have been randomized. At each site along the forest roads, we established five transects at least 50 m apart, with each transect extending 20 m from the road into the forest interior (Figure 1b). Preliminary measurements indicated that the mean temperature and relative humidity of the forest interior were nearly constant when the distance from the forest edge was 20 m or greater (Figure S1). Therefore, this transect length was considered sufficient to investigate the relationship between the physical condition of the environment and the ant community along the environmental gradient.

In each transect, we set five survey points at 5‐m intervals (0, 5, 10, 15, and 20 m inside the forest; Figure 1b; Table S1), with a total of 25 survey points at each study site. We collected ants by placing a pitfall trap and two food bait traps at each survey point. We used plastic vials (*h* = 14 cm, *φ* = 50.7 mm) containing approximately 50 ml of ethylene glycol with 2% formaldehyde as pitfall traps. Each vial was buried in the ground with its top edge level with the ground surface. The opening of each vial was covered with a plastic roof to avoid flooding by the rain. One week later, we collected the pitfall traps and immediately placed two food bait traps at each point. Each food bait trap comprised aluminum foil (6 × 6 cm) with 0.5 g of tuna and honey mixture (2:3 w/w). One food bait trap was placed on the ground and the other was placed on a tree approximately 2 m from the ground on the tree trunk closest to the study point. After 30 min, the bait traps were collected. Ants attracted to the tree traps were collected using an aspirator, whereas all the ground bait traps were quickly and carefully placed together into a plastic bag along with the ants using tweezers. These procedures enabled us to collect both the dominant and subordinate ants that were attracted to the bait (Suwabe et al., 2009). All the captured ants were identified in the laboratory using the Japanese Ant Database 2003. The species were categorized as native and exotic ants according to the antmap.org (Guénard et al., 2017) database (Table S2). During the bait‐trapping process, the disturbance involved in collecting the traps made it difficult to precisely count the number of individuals of each species, so only the presence or absence of species was recorded.

### 2.2 Effects of forest thinning on environmental conditions and the ant community

We also monitored temporal changes in the ant community in the same areas before and after the disturbance, with a focus on a deforestation project undertaken by the local government. The forest areas studied comprised an old forest stand that had not been widely cut for more than 50 years. For the thinning plot, we chose a site that had been thinned by the government in June 2005. The thinning had been undertaken in accordance with the thinning procedure guidelines of the Okinawa Prefecture. This involved selective removal of slow‐growing understory trees with a diameter at breast height of less than 3 cm. A potential problem with this public works project has been highlighted (Itô et al., 2000). We had no additional thinning plots to include in the study because the management of the areas selected for the study was controlled by the local government. Six control plots (non‐thinning) were chosen in the same forest at distances from 1.5 to 13.8 km away from the thinning plot (Figure 1a and Table S1). In the thinning plot, four transects of 30 m in length were established in the forest interior (approximately 20 m from a paved road) at 10‐m intervals. Seven survey points were set along each transect at 5‐m intervals (total *N* = 28 survey points). In each control plot, we set a 20‐m transect along which five survey points were established at 5‐m intervals (total *N* = 30 survey points in six plots). The ant community was surveyed using a pitfall trap and two bait traps using the same methods as the roadside transect study. Data were collected once before thinning (June 2005) and three times after thinning (November 2006, 2007, and 2012). We set a total of 28 pitfalls and 56 bait traps in the thinning plot and 30 pitfall traps and 60 bait traps in the control plot each year. Given that we had only one thinning plot, the data were summarized in a table, but statistical testing was avoided.

### 2.3 Measurements of environmental factors

Temperature and relative humidity were measured approximately 30 cm above the ground at each survey point from around 10:00 to 14:00 on sunny days. For the roadside transect study, measurements were undertaken twice at each point, in June 2005 and October 2006. In the study of the effects of forest thinning on environmental conditions and the ant community, we performed the same measurements for each time step (June 2005, November 2006, 2007, and 2012).

### 2.4 Statistical analyses

#### 2.4.1 Changes in the abiotic environment after road construction

To analyze the effects of distance and road age on abiotic environmental factors, we constructed linear models (LMs). We set temperature or relative humidity as the response variable, and distance, road age, and their interaction as explanatory variables. The sampling year was set as a block factor.

### 2.5 Roadside transects

We checked the spatial autocorrelation between the adjacent transects within each study site. Using all the combinations for two adjacent transects (*N* = 5; see also the horizontal lines in Figure 1b), we paired two survey points in the same distance from the road (*N* = 25). To evaluate autocorrelation, we separated the ant community by the year, site, and ant category (Tables S3 and S4), obtaining 24 communities. We focused on two types of species richness, namely the number of ant species collected by the two bait traps and pitfall trap at a survey point, and ant abundance, namely the number of ant individuals collected by the pitfall trap at a survey point. Using each value from the ant community, we performed Kendall rank correlation analyses. For the species richness of exotic ants, we detected a statistically significant positive correlation at one site in data from 2006 collected using the bait traps. Therefore, the study site (Iji) was excluded from the species richness analysis (Tables S3 and S4). For the remainder of the data, we regarded each survey point as the unit of the sample. For ant abundance, no statistically significant correlation was detected (Table S4). Therefore, when regarding each survey point as the sample unit, all the data were used for the analysis. To elucidate the factors that affect species richness and ant abundance, we applied a general linear model (GLM). This analysis was performed separately for native and exotic ant species for each trap type. The response variable was species richness or ant abundance, and the explanatory variables were road age, distance from the road, and the interaction between road age and distance. We set the sampling year as a block factor. We also evaluated the site‐specific effect of distance from the road on species richness and abundance by constructing other models. We had two sites for each road of the same age and the transects were spatially clustered according to these sites. The response variable was abundance or species richness, and the explanatory variables were site, distance from the road, the interaction between site and distance, with year as a block factor. We focused only on the interaction between the site and distance from the road.

For the statistical analyses, we checked the overdispersion of the models using function *testDispersion* in the DHARMa package (Hartig, 2020). All the effect sizes and pseudo *R* ^2^ values for the GLM (Nagelkerke, 1991) were calculated using the *r.squaredLR* function in the MuMIn package (Bartoń, 2020). The fixed effects of the models were tested using a Type II ANOVA, and *p* values were calculated based on the *χ* ^2^ values. All the statistical analyses were performed using R ver. 3.5.0 (R Development Core Team, 2010).

## 3 RESULTS

### 3.1 Relationship between the abiotic environment and the road age

**FIGURE 2:**
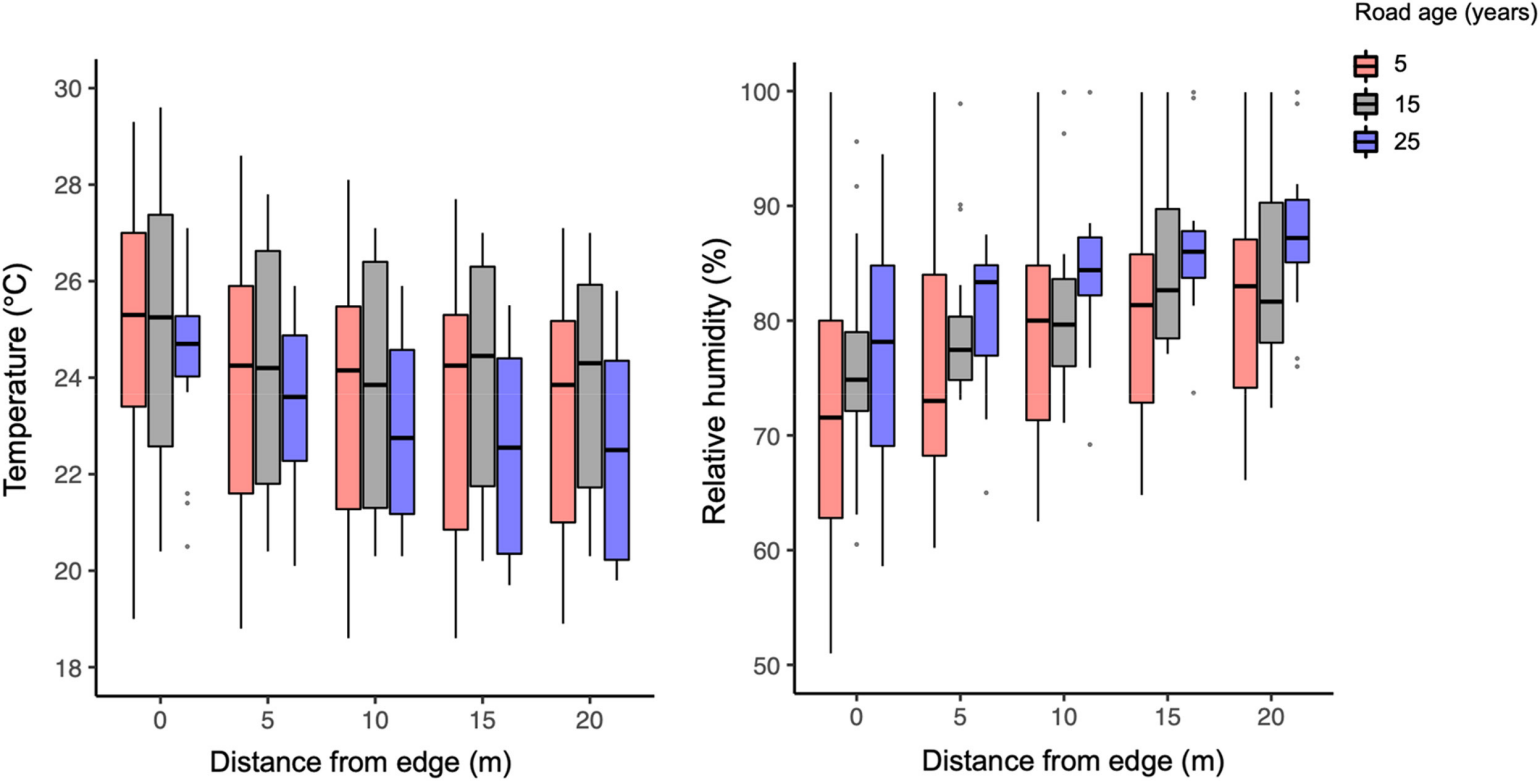
(a) Temperature and (b) relative humidity in the roadside environments and their association with time elapsed (years) since the road construction and with distance from the road edge

Temperature and relative humidity varied with distance from the road and with the age of the road. The temperature at 30 cm above the ground significantly declined with increasing distance from the road and road age (LM; *χ* ^2^ = 42.57, *p* < .001; Figure 2a). The temperature of the 25‐year‐old roadside was significantly lower than that of the 15‐ and 5‐year‐old roadsides (*χ* ^2^ = 42.57, *p* < .001; Figure 2a). The interaction between the distance and road age was not statistically significant (*χ* ^2^ = 0.90, *p* = .344; Figure 2a). Relative humidity significantly increased with increasing distance from the road and was higher for the 15‐ and 25‐year‐old roadsides than the 5‐year‐old roadside (distance: *χ* ^2^ = 54.092, *p* < .001; road age: *χ* ^2^ = 13.614, *p* < .001; Figure 2b). However, this interaction was not statistically significant (*χ* ^2^ = 0.052, *p* = .820). These results suggest that the disturbance caused by road construction made the forest floor environments at the roadsides dryer and warmer for at least 15 years after road construction. At 25 years after road construction, the roadside environments were recovering, once again becoming cooler and more humid, except immediately adjacent to the road. All the statistical values are summarized in Table S5.

### 3.2 Effects of distance from the forest edge and number of years since road construction on ant species richness

A total of 46 ant species, belonging to seven subfamilies and 28 genera, were collected using pitfall and bait traps that were set on the ground and tree trunks along the roadside transects. They comprised nine exotic and 34 native ant species (Table S2).

**FIGURE 3:**
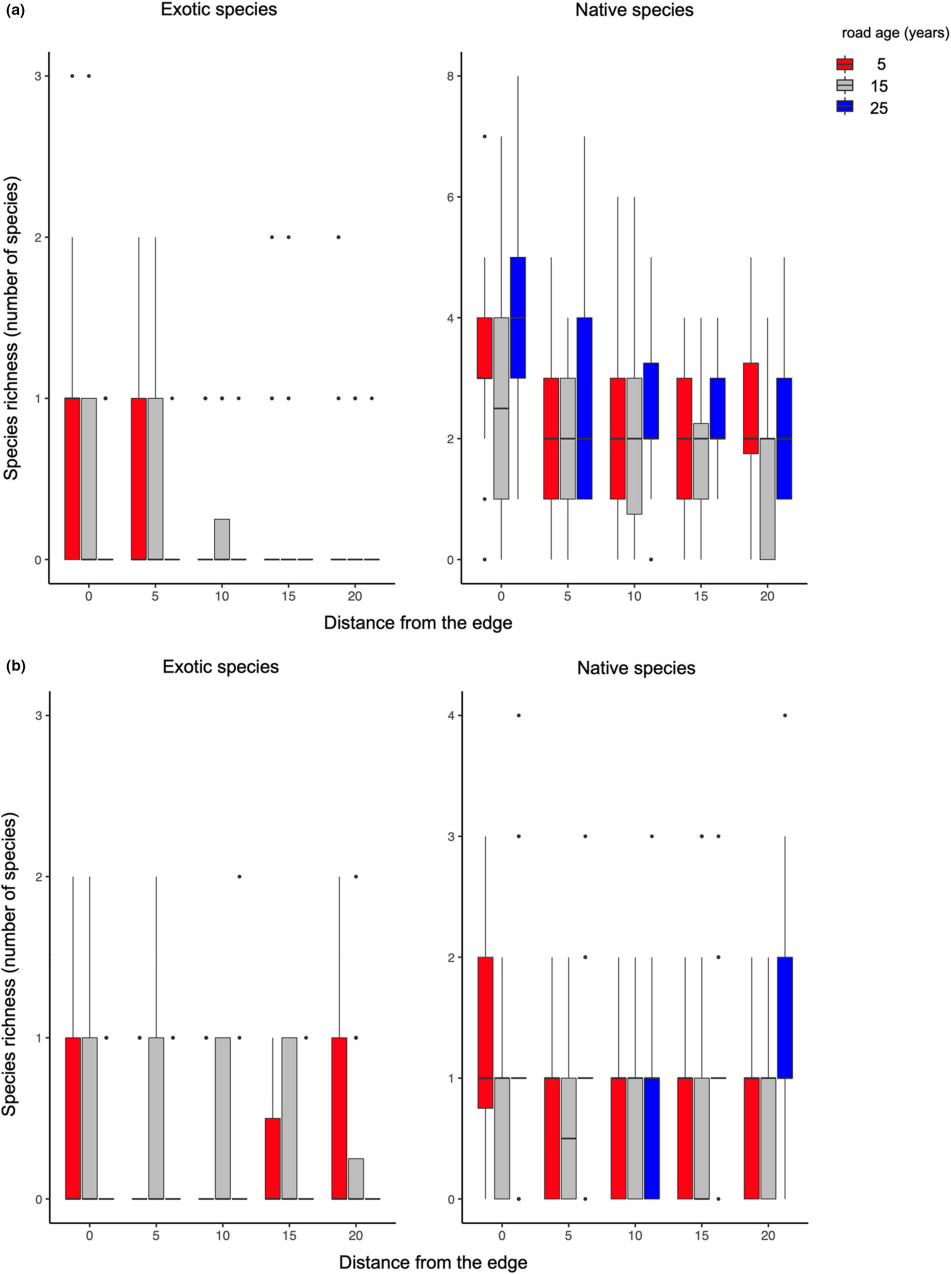
Effects of elapsed time (years) since the road construction and the distance from the road edge on species richness of exotic and native species. The species richness measurements by (a) pitfall traps and (b) bait traps were presented separately

We analyzed the effect of road age and distance from the road on species richness using pitfall trap data. The species richness of the exotic ants was significantly lower for the old road (25 years old) than for the younger roads (5 and 15 years old) (GLM with Poisson error distribution; age: *χ* ^2^ = 22.472, *p* < .001; Figure 3a). Distance from the road had a negative effect on the richness of exotic species, with more exotic species closer to the edge (*χ* ^2^ = 16.632, *p* < .001; Figure 3a). In the forest interior (20 m away from the road) of the old road (25‐year‐old) sites, almost no exotic ants were found. However, the interaction effect of distance from the road and road age on exotic species richness was not significant (*χ* ^2^ = 0.097, *p* = .755; Figure 3a). The species richness of exotic ants was higher for the 2006 survey than for the 2005 survey (*χ* ^2^ = 6.123, *p* = .013). The species richness of native ants was negatively affected by distance from the road, with more species closer to the edge than in the forest interior (GLM with Poisson distribution: distance: *χ* ^2^ = 22.326, *p* < .001; Figure 3a). The road age and interaction effects of these factors were non‐significant for native species (road age: *χ* ^2^ = 1.634, *p* = .201; interaction: *χ* ^2^ = 1.162, *p* = .281; Figure 3a). Species richness of native ants was significantly greater in 2005 than in 2006 (*χ* ^2^ = 25.085, *p* < .001).

We performed a similar analysis for species richness using the bait trap data. Almost no exotic ants were detected on the 25‐year‐old road, and the species richness of exotic ants was negatively correlated with road age (GLM with Poisson error distribution; age: *χ* ^2^ = 6.425, *p* = .011; Figure 3b). Neither the distance from the road nor its interaction with road age had a significant effect on the species richness of exotic ants (distance: *χ* ^2^ = 2.953, *p* = .086; interaction: *χ* ^2^ = 1.975, *p* = .160; Figure 3b). In contrast to the pitfall trap data, the survey year had no effect on the species richness of the exotic ants (*χ* ^2^ = 1.683, *p* = .195). The species richness of native ants had a statistically significant positive correlation with road age, but did not correlate with the distance from the road (GLM with Poisson distribution: distance: *χ* ^2^ = 0.360, *p* = .548; age: *χ* ^2^ = 4.975, *p* = .026; interaction: *χ* ^2^ = 1.299, *p* = .254; Figure 3b). More ant species were captured using bait traps in 2005 than in 2006 (*χ* ^2^ = 25.109, *p* < .001). No significant site‐specific effects of distance from the road on species richness were detected (pitfall trap: exotic: *χ* ^2^ = 5.846, *p* = .321; native: *χ* ^2^ = 6.272, *p* = .281; Bait trap: exotic: *χ* ^2^ = 5.305, *p* = 0.380; native: *χ* ^2^ = 1.955, *p* = .855). The statistical values for these analyses are summarized in Tables S6 and S7. The statistics for the site‐specificity analysis are shown in this text but not in the table.

In summary, more exotic ant species were found on the roadsides of young roads than on old roads, and the native ants had the opposite tendency, which was more pronounced for the bait trap data. Species richness was higher in the vicinity of the road than in the forest interior, regardless of whether the ants were native or exotic. This was with the exception of the native ant species richness data in the bait traps that had no significant correlation with distance from the road.

### 3.3 Effects of distance from forest edge and the number of years since road construction on ant abundance

**FIGURE 4:**
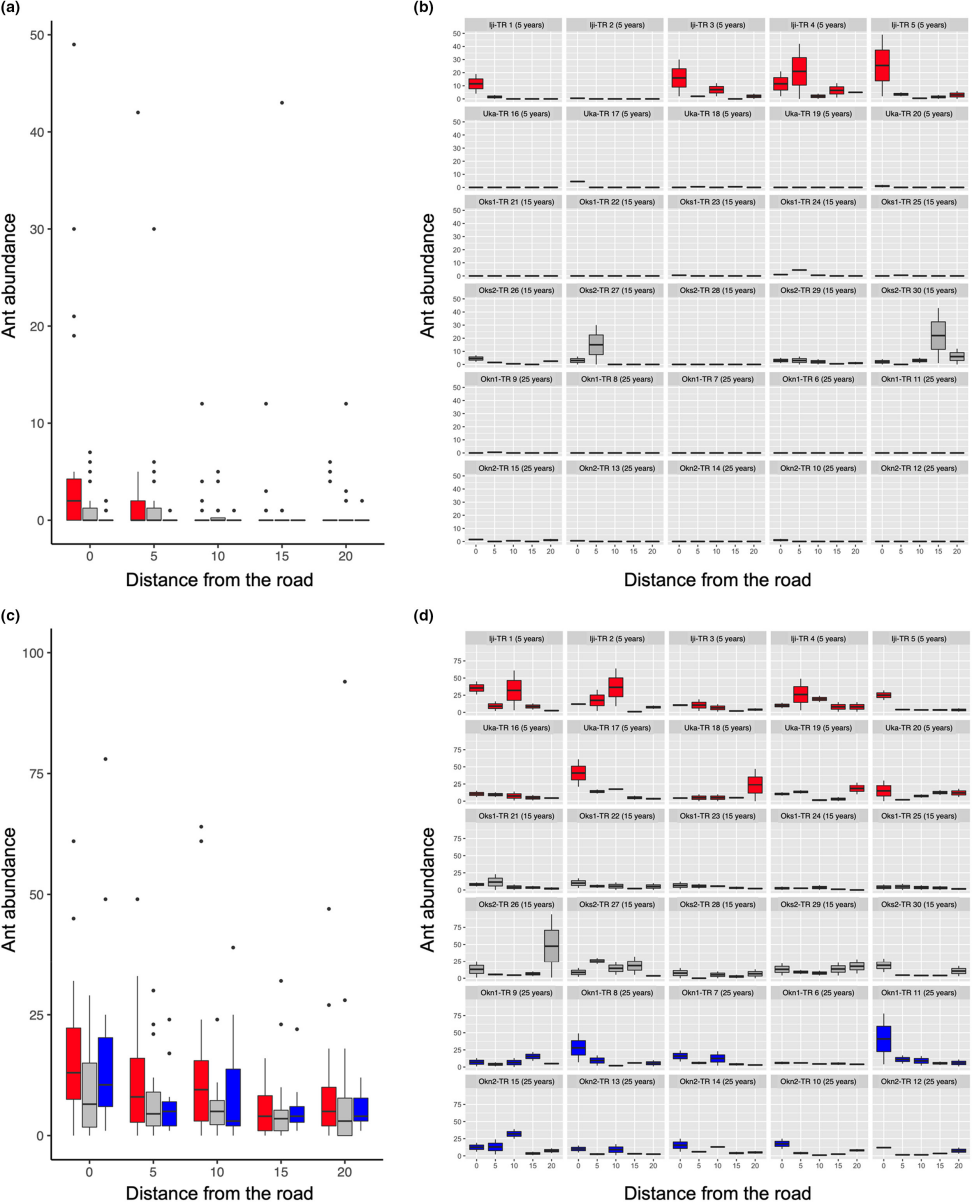
Effects of elapsed time (years) since the road construction and distance from the road edge on abundance of (a) exotic and (c) native species using the data from the pitfall traps. The data are represented by each site for (b) exotic and (d) native ants

For exotic ants, abundance was significantly lower for the old roads (GLM with negative binomial error distribution; road age: *χ* ^2^ = 23.082, *p* < .001; distance: *χ* ^2^ = 6.568, *p* = .010; Figure 4a), whereas the interaction was statistically non‐significant (*χ* ^2^ = 0.000, *p* = .998; Figure 4a). Ant abundance was significantly lower in 2005 than in 2006 (*χ* ^2^ = 14.404, *p* < .001). We found that there was no site‐specific effect (*χ* ^2^ = 8.870, *p* = .114; Figure 4b). The abundance of native ants was significantly higher at the forest edge than in the forest interior and was negatively correlated with road age (GLM with negative binomial error distribution: distance: *χ* ^2^ = 16.243, *p* < .001; age: *χ* ^2^ = 5.314, *p* = .021; Figure 4c). However, the effect of the distance–road age interaction was not statistically significant (*χ* ^2^ = 0.010, *p* = .919; Figure 4c). Native ant abundance was significantly higher in 2005 than in 2006 (*χ* ^2^ = 33.217, *p* < .001). We also found site‐specificity in the relationship with distance (*χ* ^2^ = 12.701, *p* = .026). The distance‐dependent tendencies were not found at some sites, including the 5‐ and 15‐year‐old roadsides (Figure 4d). The statistics are summarized in Table S8. The statistics for the site‐specificity analysis are shown only in this section but not in the table.

Our data indicated that exotic and native ants were more abundant in the roadside environments of younger roads, whereas the abundance of native species was higher at the forest edge than in the interior, regardless of the road age. However, for native ants, the results of the distance dependency analysis should be interpreted carefully because site‐specific effects were detected.

### 3.4 Effect of forest thinning on physical condition of the environment and on the ant species richness

After forest thinning in 2006 and 2007, we found a substantial increase in the occurrence of *T. brunneus*, which was the only exotic ant observed in the thinned plot. However, in the control plots, the occurrence of *T. brunneus* was consistently low (Table 1a). In the thinned plot, *T*. *brunneus* was found at all points in 2007. However, in 2012, it was observed at only 14 of the 28 points (Table 1a). In contrast to the population dynamics of *T*. *brunneus* in the thinning plots, the species richness and abundance of native ants were almost constant throughout the study period (Table 1a). We also monitored changes in the abiotic environment over time after thinning. Thinning changed the environment on the forest floor with an increase in temperature and a decrease in moisture availability. However, by 2012 the environment had recovered to its original state (Table 1b).

**TABLE 1:** Summary of the results of the forest thinning over time. (a) Species richness and abundance of native ants and the occurrence frequency of *Technomyrmex brunneus*. (b) Environmental factors: Our survey was carried out once before forest thinning (2005) and three times after forest thinning (2006, 2007, and 2012)

	Treatment	2005	2006	2007	2012
(a)					
Number of native species	Control plots	7.7 [7–9]^a^	6 [5–8]	6.3 [4–8]	5 [3–7]
	Thinning plots^b^	6.5 [5–9]	7 [6–9]	6.5 [5–8]	7.3 [5–9]
Abundance of native species	Control plots	92.2 [64–143]	25.2 [13–37]	22.7 [8–43]	13.5 [3–34]
	Thinning plots^b^	32.5 [21–44]	31.3 [12–49]	39 [13–75]	16.3 [10–20]
Occurrence frequency of *T*. *brunneus*	Control plots	0.20 [6/30]	0.07 [2/30]	0.13 [4/30]	0.30 [9/30]
	Thinning plots^b^	0.07 [2/28]	0.79 [22/28)	1.00 [28/28]	0.50 [14/28]
(b)					
Temparature (°C)	Control plots	17.9 [17.1–19.2]	25.1 [23.9–26.9]	23.5 [22.7–24.9]	20.8 [17.6–22.5]
	Thinning plots^b^	18.5 [17.8–19.3]	27.4 [27.0–27.7]	24.4 [24.0–25.0]	18.1 [17.8–18.5]
Relative humidty (%)	Control plots	96.9 [89.4–99.9]	82.6 [75.5–86.6]	85.6 [81.8–88.2]	73.8 [62.4–87.9]
	Thinning plots^b^	92.2 [87.1–96.6]	70.4 [67.6–75.7]	78.7 [76.7–81.5]	73 [69.6–76.2]

^a^ Mean [data range].

^b^ Data from one disturbed area.

## 4 DISCUSSION

### 4.1 Potential resilience of the native ant community

Our comparative study in the Yanbaru forests, where forest stands were older than 50 years, suggested that the construction of paved roads created warmer and drier roadside environments. The environmental gradient extended to cooler and moister forest interiors. The effects of forest disturbance on local conditions lasted for at least 15 years after road construction. However, after 25 years, the physical conditions of the roadside environments could likely recover, except the areas immediately adjacent to the road (Figure 2). Enoki et al. (2014) also compared roadside environments in Yanbaru and reported a similar recovery 20 years after road construction. The recovery of vegetation, especially through the lateral extension of branches, led to canopy reclosure. In our study, tree branches overhanging the road were only observed along roads older than 25 years old.

Ant communities also appear to be related to these environmental gradients. The species richness and potentially the abundance of exotic ants were higher in the roadside environments, especially immediately adjacent to the roadsides of young roads, where the physical effect of environmental disturbance (being warmer and drier) was prominent. However, on the roadsides of the 25‐year‐old roads, almost no exotic ants were collected (Figure 3a,b). The species richness of the native ants was not affected by road age, but was even higher at the forest edge than in the forest interior (Figure 3a,b). Experimental thinning inside and not at the edge of the forest led to an immediate “outbreak” after one or two years of the exotic ant, *T. brunneus* (Table 1). This “outbreak” seemed to have settled 7 years after thinning. During this “outbreak,” the species richness of the native ants was almost constant (Table 1). These data lead to the hypothesis that in the Yanbaru forest, native ant communities have a degree of resilience to anthropogenic disturbances such as road construction and thinning. The disturbance from the road construction led to the recruitment of exotic ants, while the native ant community showed “recovery,” a long time after the disturbance. However, our resilience hypothesis would have to be tested in studies where the same roadside environment is observed for years after the road was opened.

Our hypothesis implies that to control the spread of exotic ants in the Yanbaru forest, it is more effective to restore the habitats to their pre‐disturbance state than to directly control the exotic ant populations, using methods such as pesticides. Conservation efforts should be directed towards supporting the recovery of vegetation in roadside environments. The construction of new paved roads has to be avoided. However, we are not sure whether this conservation policy can be applied to potential future cases of unintroduced invasive ants, such as *Solenopsis invicta* and *Wasmannia auropunctata*. In such cases, appropriate measures must be carefully chosen.

### 4.2 Seasonality in the activity of exotic and native ants

We found a statistically significant effect of survey year, with more native ants being collected in June 2005 than in October 2006. The opposite was the case for the exotic ants. This does not suggest that exotic ants are increasing in place of native ants in Yanbaru during this period. This may confirm the influence of seasonality as suggested by Suwabe et al. (2009). In Yanbaru's roadside environments, native ants dominate over exotic ants in summer, and more exotic ants are trapped during spring and autumn when native ants become less active.

### 4.3 Mechanisms of disturbed habitat dependence in exotic ants

Exotic ants increased in richness and/or abundance after road construction and forest thinning, although this effect may be temporary. The disturbance dependence of exotic species was also confirmed in this study. Many authors have discussed the mechanism underlying the disturbance dependence of invasive species, including ants (see Section 1). Although the precise mechanism remains unclear, our results provide some insights. The native ant species did not decrease in the disturbed environment where exotic ants were common. The absence of a negative relationship suggests that the removal of the dominant native competitors is irrelevant to the observed prevalence of exotic ants in disturbed habitats.

A potential mechanism is that anthropogenic disturbance modifies the physical environment to be more suitable for exotic ants. The high temperature and low humidity in the disturbed habitats may improve conditions for exotic species to thrive. Among the exotic species observed, *Paratrechina longicoronis*, *Pheidole fervens*, *Anoplolepis gracilipes*, *Tetramorium bicarinatum*, *Cardiocondyla kagutsuchi*, *Nylanderia amia*, and *Pheidole parva* originated in more tropical regions at lower latitudes than at Okinawa (Table 2).

**TABLE 2:** Ecological characteristics of exotic ants found in the study

Species	Native region	Distributional region	Gyny	Colony foundation	Colony structure	Reference
*Pheidole parva*	Indomalaya	Palearctic, Indomalaya, Oceania, Malagasy	–	–	–	
*Pheidole fervens*	Indomalaya, Australasia	Palearctic, Nearctic, Australasia, Indomalaya	Polygyny	–	Polydomous	Sarnat et al. (2015)
*Tetramorium bicarinatum*	Indomalaya	Palearctic, Nearctic, Afrotropical, Neotropic, Australasia, Indomalaya	Polygyny	–	Polydomous	Astruc et al. (2001)
*Tetramorium kraepelini*	–	Palearctic, Indomalaya	–	–	–	
*Tetramorium kraepelini*	Indomalaya	Palearctic, Afrotropical, Neotropic, Australasia, Indomalaya, Oceania, Malagasy	Polygyny	Fucultative	Supercolonial	Thomas et al. (2010)
*Paratrechina longicornis*	Afrotropical	Palearctic, Nearctic, Afrotropical, Neotropic, Australasia, Indomalaya	Polygyny	Dependent	Polydomous	Yamauchi and Ogata (1995)
*Nylanderia amia*	–	Palearctic, Indomalaya	–	–	–	
*Technomyrmex brunneus*	Indomalaya	Palearctic, Afrotropical, Neotropic, Australasia, Indomalaya, Oceania, Malagasy	Polygyny	Fucultative	Polydomous	Yamauchi et al. (1991)

Another potential mechanism is that the life history or behavioral characteristics shared by exotic species promote their prevalence in disturbed habitats through the interaction between traits and disturbance. In addition to dwelling in disturbed habitats, several other traits are found more frequently in exotic ant species than in native species (Bertelsmeier et al., 2017; Passera, 1994). Polygyny increases the chance of a colony propagule containing a reproductive queen (Buczkowski, 2010; Hölldobler & Wilson, 1977; Linksvayer & Janssen, 2009), leading to a higher probability of colony founding by chance (dependent colony founding). Tsuji and Tsuji (1996) developed a mathematical model that proposed another general ecological advantage of dependent colony founding in frequently disturbed habitats, namely faster population growth. The advantages of dependent colony founding can exceed costs, such as local saturation due to limited dispersal, under a specific disturbance regime (Nakamaru et al., 2007, 2014).

### 4.4 Resource competition and disturbance

At the forest edge, the richness of native species was not negatively associated with the richness of exotic species. The pitfall trapping data suggested that the overall abundance of ants increased at the forest edge (Figure 4a,c).

Heterogeneity in physical environments (Lawton, 1983) created by the presumed intermediate‐level disturbance due to road construction (Connell, 1978) may have led to the coexistence of more ant species at the forest edge. The enrichment of resources may have also been related to this change. At roadsides, the light conditions on the forest floor are improved (Griffis et al., 2001; Thomas et al., 1999), allowing vegetation such as grass and herbs to increase the biomass and diversity (Thibodeau et al., 2000; Weng et al., 2007). In ecosystems dominated by herbaceous plants, the flow of primary production to herbivory is greater than that in forests dominated by woody plants (Begon et al., 2006). As a result, the richness and abundance of arthropods at the forest edge may have increased, leading to an increase in the abundance and diversity of ants hunting these arthropods (Maleque et al., 2007a, 2007b; Ohsawa, 2004; Taki et al., 2010). Within the lower vegetation along the roadsides of Yanbaru, the availability of food resources from honeydew‐producing insects (Tanaka et al., 2011) and extrafloral nectary plants (Katayama & Tsuji, 2010) is higher than that on the floor of the forest interior, where the stand has developed over a longer time (Tanaka et al., 2019). These food resources are consumed by exotic (Styrsky & Eubanks, 2006; Tanaka et al., 2011) and native ants (Katayama & Tsuji, 2010), which may have positively affected the diversity and abundance of the exotic ants (Helms & Vinson, 2002; Helms & Vinson, 2008) and ants in general (Davidson, 1997; Davidson et al., 2003).

### 4.5 Ant dispersal strategies and anthropogenic disturbance

Among the nine exotic ants, *T. brunneus* occurred relatively frequently inside the forest (Table S2). This species is native to southern Asia and has recently expanded its distribution north to many islands, showing invasiveness on some islands such as Hachiojima (Putri et al., 2021). Although the survey sample size was small (one site for forest thinning; see Section 2), an outbreak of *T*. *brunneus* occurred at the thinning site (Table 1). This difference from other exotic ants, which were found only on roadsides, likely reflects the dispersal strategies of *T*. *brunneus*. As noted, *T*. *brunneus* can perform independent and dependent colony formation. Alates can disperse over long distances and can, therefore, find a colony in the forest interior. Nests of *T*. *brunneus* are often found in the middle of the forest in dry, dead wood, especially in naturally disturbed habitats such as gaps or windy ridges (K. Tsuji, personal observation). These isolated colonies in the forest interior were highly likely to have been found with alate queens. When the forest was disturbed by thinning, it is likely that these small artificial gaps were quickly occupied by *T*. *brunneus* through budding from a prior existing colony with low abundance in the middle of the forest. Quick budding is easy for mature *T*. *brunneus* colonies because these nests contain many wingless reproductives (Yamauchi et al., 1991). The other exotic ants are all polygynous and polydomous, and their main mode of colony foundation is most likely dependent founding, although they have alate queens. Because they are only dispersed on foot, they are likely unable to reach an isolated gap deep in the forest. Instead, they expand their distribution along roadside disturbed habitats that are suitable for exotic ants to flourish. Corridors that connect suitable habitats for exotic ants can aid in their spread (Holway, 2005; Resasco et al., 2014). The paved roads act as spatially connected corridors for exotic ants to facilitate the expansion of their distribution. It is vital that further construction of paved roads is avoided in the Yanbaru Forest.

## AUTHOR CONTRIBUTIONS

**Mayuko Suwabe:** Investigation (lead); methodology (equal). **Tomonori Kikuchi:** Investigation (equal); methodology (equal). **Hitoshi Ohnishi:** Investigation (equal); methodology (equal). **Hirotaka Tanaka:** Investigation (equal). **Kengo Kawara:** Investigation (equal). **Yusuke Hidaka:** Investigation (equal). **Tsutomu Enoki:** Conceptualization (equal); project administration (equal). **Kazuki Tsuji:** Conceptualization (lead); funding acquisition (lead); project administration (lead); supervision (lead); writing – original draft (lead); writing – review and editing (lead). **Hiroyuki Shimoji:** Data curation (lead); formal analysis (lead); funding acquisition (supporting); methodology (equal); visualization (lead); writing – original draft (equal); writing – review and editing (equal).

## CONFLICT OF INTEREST

The authors declare no competing interests.

## Supporting information


Appendix S1

Appendix S2

Appendix S3

Appendix S4

Appendix S5

Appendix S6

Appendix S7

Appendix S8

Appendix S9

Appendix S10

Supplementary Material

Appendix S11

## Data Availability

All data and R script used to analyses in this study is deposited in the Supplementary materials in this journal.

## References

[ece39073-bib-0001] Abe, T. , Kudo, T. , Saito, K. , Takashima, T. , & Miyamoto, A. (2021). Plant indicator species for the conservation of priority forest in an insular forestry area, Yambaru, Okinawa Island. Journal of Forest Research, 26(3), 181–191. 10.1080/13416979.2020.1858535

[ece39073-bib-0002] Arnan, X. , Andersen, A. N. , Gibb, H. , Parr, C. L. , Sanders, N. J. , Dunn, R. R. , Angulo, E. , Baccaro, F. B. , Bishop, T. R. , Boulay, R. , Castracani, C. , Cerdá, X. , Toro, I. D. , Delsinne, T. , Donoso, D. A. , Elten, E. K. , Fayle, T. M. , Fitzpatrick, M. C. , Gómez, C. , … Retana, J. (2018). Dominance‐diversity relationships in ant communities differ with invasion. Global Change Biology, 24, 4614–4625. 10.1111/gcb.14331 29851235

[ece39073-bib-0003] Astruc, C. , Malosse, C. , & Errard, C. (2001). Lack of intraspecific aggression in the ant *Tetramorium bicarinatum*: a chemical hypothesis. Journal of Chemical Ecology, 27, 1229–1248. 10.1023/a:1010324230982 11504025

[ece39073-bib-0004] Bartoń, K. (2020). MuMIn: Multi‐model inference . R package version 1.43.17. https://CRAN.R‐project.org/package=MuMIn

[ece39073-bib-0005] Bauer, J. T. (2012). Invasive species: “back‐seat drivers” of ecosystem change? Biological Invasions, 14, 1295–1304. 10.1007/s10530-011-0165-x

[ece39073-bib-0006] Begon, M. , Townsend, C. R. , & Harper, J. (2006). Ecology: From individuals to ecosystems (4th ed.). Blackwell Publishing Ltd.

[ece39073-bib-0007] Bellard, C. , Cassey, P. , & Blackburn, T. M. (2016). Alien species as a driver of recent extinctions. Biology Letters, 12, 20150623. 10.1098/rsbl.2015.0623 26888913 PMC4780541

[ece39073-bib-0008] Bertelsmeier, C. , Ollier, S. , Liebhold, A. , & Keller, L. (2017). Recent human history governs global ant invasion dynamics. Nature Ecology and Evolution, 1, 184. 10.1038/s41559-017-0184 PMC5495171 28685166

[ece39073-bib-0009] Blumenfeld, A. J. , Eyer, P.‐A. , Helms, A. M. , Buczkowski, G. , & Vargo, E. L. (2021). Consistent signatures of urban adaptation in a native, urban invader ant *Tapinoma sessile* . Molecular Ecology. 10.1111/mec.16188 34551170

[ece39073-bib-0010] Buczkowski, G. (2010). Extreme life history plasticity and the evolution of invasive characteristics in a native ant. Biological Invasions, 12, 3343–3349. 10.1007/s10530-010-9727-6

[ece39073-bib-0011] Connell, J. H. (1978). Diversity in tropical rain forests and coral reefs. Science, 199, 1302–1310. 10.1126/science.199.4335.1302 17840770

[ece39073-bib-0012] Davidson, D. W. (1997). The role of resource imbalances in the evolutionary ecology of tropical arboreal ants. Biological Journal of the Linnean Society, 61, 153–181. 10.1111/j.1095-8312.1997.tb01785.x

[ece39073-bib-0013] Davidson, D. W. , Cook, S. C. , Snelling, R. R. , & Chua, T. H. (2003). Explaining the abundance of ants in lowland tropical rainforest canopies. Science, 300, 969–972. 10.1126/science.1082074 12738862

[ece39073-bib-0014] Didham, R. K. , Tylianakis, J. M. , Hutchison, M. A. , Ewers, R. M. , & Gemmell, N. J. (2005). Are invasive species the drivers of ecological change? Trends in Ecology and Evolution, 20, 470–474. 10.1016/j.tree.2005.07.006 16701420

[ece39073-bib-0015] Elton, C. S. (1958). The ecology of invasions by animals and plants. Methuen.

[ece39073-bib-0016] Enoki, T. , Kusumoto, B. , Igarashi, S. , & Tsuji, K. (2014). Stand structure and plant species occurrence in forest edge habitat along different aged roads on Okinawa Island, southwestern Japan. Journal of Forest Research, 19, 97–104. 10.1007/s10310-012-0383-9

[ece39073-bib-0017] Foucaud, J. , Orive, J. , Loiseau, A. , Delabie, J. H. C. , Jourdan, H. , Konghouleux, D. , Vonshak, M. , Tindo, M. , Mercier, J.‐L. , Fresneau, D. , Mikissa, J.‐B. , McGlynn, T. , Mikheyev, A. S. , Oettler, J. , & Estoup, A. (2010). Worldwide invasion by the little fire ant: routes of introduction and eco‐evolutionary pathways. Evolutionary Application, 3, 363–374. 10.1111/j.1752-4571.2010.00119.x PMC3352468 25567931

[ece39073-bib-0018] Gause, G. F. (1935). Verifications experimentales de la theorie mathematique de al lutte pour la vie. In Actualites Scientifues et Industrielles (p. 227). Hermann.

[ece39073-bib-0019] Goodman, M. , & Warren, R. J., II . (2019). Non‐native ant invader displaces native ants but facilitates non‐predatory invertebrates. Biological Invasions, 21, 2713–2722. 10.1007/s10530-019-02005-w

[ece39073-bib-0020] Griffis, K. L. , Crawford, J. A. , Wagner, M. R. , & Moir, W. H. (2001). Understory response to management treatments in northern Arizona ponderosa pine forests. Forest Ecology and Management, 146, 239–245. 10.1016/S0378-1127(00)00461-8

[ece39073-bib-0021] Guénard, B. S. , Weiser, M. D. , Gómez, K. , Narula, N. , & Economo, E. P. (2017). The Global Ant Biodiversity Informatics (GABI) database: synthesizing data on the geographic distribution of ant species (Hymenoptera: Formicidae). Myrmecological News, 24, 83–89. 10.25849/myrmecol.news_024:083

[ece39073-bib-0022] Gurevitch, J. , & Padilla, D. K. (2004). Are invasive species a major cause of extinctions? Trends in Ecology and Evolution, 19, 470–474. 10.1016/j.tree.2004.07.005 16701309

[ece39073-bib-0023] Hackerott, S. , Valdivi, A. , Green, S. J. , Côté, I. M. , Cox, C. E. , Akins, L. , Layman, C. A. , Precht, W. F. , & Bruno, J. F. (2013). Native predators do not influence invasion success of pacific lionfish on Caribbean reefs. PLoS One, 8, e68259. 10.1371/journal.pone.0068259 23874565 PMC3708960

[ece39073-bib-0024] Hartig, F. (2020). DHARMa: Residual diagnostics for hierarchical (multi‐level/mixed) regression models . R package version 0.3.3.0. https://CRAN.R‐project.org/package=DHARMa

[ece39073-bib-0025] Helms, K. R. , & Vinson, S. B. (2002). Widespread association of the invasive ant *Solenopsis invicta* with an invasive mealybug. Ecology, 83, 2425–2438. 10.1890/0012-9658(2002)083[2425:WAOTIA]2.0.CO;2

[ece39073-bib-0026] Helms, K. R. , & Vinson, S. B. (2008). Plant resources and colony growth in an invasive ant: The importance of honeydew‐producing Hemiptera in carbohydrate transfer across trophic levels. Environtal Entomology, 37, 487–493. 10.1603/0046-225x(2008)37[487:pracgi]2.0.co;2 18419921

[ece39073-bib-0027] Hölldobler, B. , & Wilson, E. O. (1977). The number of queens: An important trait in ant evolution. Die Naturwissenschaften, 64, 8–15. 10.1007/BF00439886

[ece39073-bib-0028] Hölldobler, B. , & Wilson, E. O. (1990). The ants. The Belknap Press of Harvard University Press.

[ece39073-bib-0029] Holway, D. A. (2005). Edge effects of an invasive species across a natural ecological boundary. Biological Conservation, 121, 561–567. 10.1016/j.biocon.2004.06.005

[ece39073-bib-0030] Holway, D. A. , Lach, L. , Suarez, A. V. , Tsutsui, N. D. , & Case, T. J. (2002). The causes and consequences of ant invasions. Annual Review of Ecology, Evolution, and Systematics, 33, 181–233. 10.1146/annurev.ecolsys.33.010802.150444

[ece39073-bib-0031] Itô, Y. , Miyagi, K. , & Ota, H. (2000). Imminent extinction crisis among the endemic species of the forests of Yanbaru, Okinawa, Japan. Oryx, 34, 305–316.

[ece39073-bib-0032] Jauni, M. , Gripenberg, S. , & Ramula, S. (2015). Non‐native plant species benefit from disturbance: a meta‐analysis. Oikos, 124, 122–129. 10.1111/oik.01416

[ece39073-bib-0033] Katayama, M. , & Tsuji, K. (2010). Habitat differences and occurrence of native and exotic ants on Okinawa Island. Entomological Science, 13, 425–429. 10.1111/j.1479-8298.2010.00400.x

[ece39073-bib-0034] King, J. R. , & Tschinkel, W. R. (2008). Experimental evidence that human impacts drive fire ant invasions and ecological change. Proceedings of the National Academy of Sciences of the United States of America., 105, 20339–20343. 10.1073/pnas.0809423105 19064909 PMC2629336

[ece39073-bib-0035] King, J. R. , & Tschinkel, W. R. (2013). Experimental evidence for weak effects of fire ants in a naturally invaded pine‐savanna ecosystem in north Florida. Ecological Entomology., 38, 68–75. 10.1111/j.1365-2311.2012.01405.x

[ece39073-bib-0036] Lawton, J. H. (1983). Plant architecture and the diversity of phytophagous insects. Annual Review of Entomology, 28, 23–39. 10.1146/annurev.en.28.010183.000323

[ece39073-bib-0037] LeBrun, E. G. , Plowes, R. M. , & Gilbert, L. E. (2012). Important fire ants near the edge of their range: disturbance and moisture determine prevalence and impact of an invasive social insect. Journal of Animal Ecology, 81, 884–895. 10.1111/j.1365-2656.2012.01954.x 22292743

[ece39073-bib-0038] Linksvayer, T. A. , & Janssen, M. A. (2009). Traits underlying the capacity of ant colonies to adapt to disturbance and stress regimes. Systems Research and Behavioral Science, 26, 315–329. 10.1002/sres.928

[ece39073-bib-0039] MacDougall, A. S. , & Turkington, R. (2005). Are invasive species the drivers or passengers of change in degraded ecosystem? Ecology, 86, 42–55. 10.1890/04-0669

[ece39073-bib-0040] Maleque, M. A. , Ishii, H. T. , Maeto, K. , & Taniguchi, S. (2007a). Line thinning enhances diversity of Coleoptera in overstocked *Cryptomeria japonica* plantations in central Japan. Arthropod‐Plant Interactions, 1, 175–185. 10.1007/s11829-007-9016-1

[ece39073-bib-0041] Maleque, M. A. , Ishii, H. T. , Maeto, K. , & Taniguchi, S. (2007b). Line thinning fosters the abundance and diversity of understory Hymenoptera (Insecta) in Japanese cedar (*Cryptomeria japonica* D. Don) plantations. Journal of Forest Research, 12, 14–23. 10.1007/s10310-006-0243-6

[ece39073-bib-0042] Menke, S. B. , Ward, P. S. , & Holway, D. A. (2018). Long‐term record of Argentine ant invasions reveals enduring ecological impacts. Ecology, 99, 1194–1202. 10.1002/ecy.2200 29504667

[ece39073-bib-0043] Mitchell, C. E. , & Power, A. G. (2003). Release of invasive plants from fungal and viral pathogens. Nature, 421, 625–627. 10.1038/nature01317 12571594

[ece39073-bib-0044] Murray, B. R. , & Phillips, M. L. (2010). Investment in seed dispersal structures is linked to invasiveness in exotic plant species of south‐eastern Australia. Biological Invasions, 12, 2265–2275. 10.1007/s10530-009-9637-7

[ece39073-bib-0045] Nagelkerke, N. J. D. (1991). A note on a general definition of the coefficient of determination. Biometrika, 78, 691–692. 10.1093/biomet/78.3.691

[ece39073-bib-0046] Nakamaru, M. , Beppu, Y. , & Tsuji, K. (2007). Does disturbance favor dispersal? An analysis of ant migration using the colony‐based lattice model. Journal of Theoretical Biology, 248, 288–300. 10.1016/j.jtbi.2007.05.012 17583750

[ece39073-bib-0047] Nakamaru, M. , Takada, T. , Ohtsuki, A. , Suzuki, S. U. , Miura, K. , & Tsuji, K. (2014). Ecological conditions favoring budding in colonial organisms under environmental disturbance. PLoS One, 9, e91210. 10.1371/journal.pone.0091210 24621824 PMC3951312

[ece39073-bib-0048] Ohsawa, M. (2004). Species richness of Cerambycidae in larch plantations and natural broad‐leaved forests of the central mountainous region of Japan. Forest Ecology and Management, 189, 375–385. 10.1016/j.foreco.2003.09.007

[ece39073-bib-0049] Passera, L. (1994). Characteristics of tramp species. In D. F. Williams (Ed.), Exotic ants: Biology, impacts and control of introduced species (pp. 23–43). Boulder.

[ece39073-bib-0050] Putri, D. , Yokozawa, M. , Yamanaka, T. , & Cronin, A. L. (2021). Trait plasticity among invasive populations of the ant *Technomyrmex brunneus* in Japan. Animals, 11, 2702. 10.3390/ani11092702 34573668 PMC8465827

[ece39073-bib-0051] R Development Core Team . (2010). R: A language and environment for statistical computing. R Foundation for Statistical Computing.

[ece39073-bib-0052] Resasco, J. , Haddad, N. M. , Orrock, J. H. , Shoemaker, D. W. , Brudvig, L. A. , Damschen, E. I. , Tewksbury, J. T. , & Levey, D. J. (2014). Landscape corridors can increase invasion by an exotic species and reduce diversity of native species. Ecology, 95, 2033–2039. 10.1890/14-0169.1 25230454

[ece39073-bib-0053] Roeder, K. A. , Useche, V. P. , Levey, D. J. , & Resasco, J. (2021). Testing effects of invasive fire ants and disturbance on ant communities of the longleaf pine ecosystem. Ecological Entomology, 46, 964–972. 10.1111/een.13033

[ece39073-bib-0054] Roura‐Pascual, N. , Hui, C. , Ikeda, T. , Leday, G. , Richardson, D. M. , Carpintero, S. , Espadaler, X. , Gómez, C. , Guénard, B. , Hartley, S. , Krushelnycky, P. , Lester, P. J. , McGeoch, M. A. , Menke, S. B. , Pedersen, J. S. , Pitt, J. P. W. , Reyes, J. , Sanders, N. J. , Suarez, A. V. , … Worner, S. P. (2010). Relative roles of climatic suitability and anthropogenic influence in determining the pattern of spread in a global invader. Proceedings of the National Academy of Sciences of the United States of America, 108, 220–225. 10.1073/pnas.1011723108 21173219 PMC3017164

[ece39073-bib-0055] Salyer, A. , Bennett, G. W. , & Buczkowski, G. A. (2014). Odorous house ants (*Tapinoma sessile*) as back‐seat drivers of localized ant decline in urban habitats. PLoS One, 9, e113878. 10.1371/journal.pone.0113878 25551819 PMC4281180

[ece39073-bib-0056] Sarnat, E. M. , Fisher, G. , Guenard, B. , & Economo, E. P. (2015). Introduced *Pheidole* of the world: taxonomy, biology and distribution. Zookeys, 543, 1–109. 10.3897/zookeys.543.6050 PMC4714327 26798286

[ece39073-bib-0057] Shea, K. , & Chesson, P. (2002). Community ecology theory as a framework for biological invasions. Trends in Ecology and Evolution, 17, 170–176. 10.1016/S0169-5347(02)02495-3

[ece39073-bib-0058] Stuble, K. L. , Kirkman, L. K. , Carroll, C. R. , & Sanders, N. J. (2011). Relative effects of disturbance on red imported fire ants and native ant species in a longleaf pine ecosystem. Conservation Biology, 25, 618–622. 10.1111/j.1523-1739.2010.01634.x 21561472

[ece39073-bib-0059] Stuhler, J. D. , & Orrock, J. L. (2016). Historical land use and present‐day canopy thinning differentially affect the distribution and abundance of invasive and native ant species. Biological Invasions, 18, 1813–1825. 10.1007/s10530-016-1122-5

[ece39073-bib-0060] Styrsky, J. D. , & Eubanks, M. D. (2006). Ecological consequences of interactions between ants and honeydew‐producing insects. Proceedings of the Royal Society. Series B: Biological Sciences, 274, 151–164. 10.1098/rspb.2006.3701 PMC1685857 17148245

[ece39073-bib-0061] Suwabe, M. , Ohnishi, H. , Kikuchi, T. , Kawara, K. , & Tsuji, K. (2009). Difference in seasonal activity pattern between non‐native and native ants in subtropical forest of Okinawa Island, Japan. Ecological Research, 24, 637–643. 10.1007/s11284-008-0534-9

[ece39073-bib-0062] Taki, H. , Inoue, T. , Tanaka, H. , Makihara, H. , Sueyoshi, M. , Isono, M. , & Okabe, K. (2010). Responses of community structure, diversity, and abundance of understory plants and insect assemblages to thinning in plantations. Forest Ecology and Management, 259, 607–613. 10.1016/j.foreco.2009.11.019

[ece39073-bib-0063] Tanaka, H. , Ohnishi, H. , Tatsuta, H. , & Tsuji, K. (2011). An analysis of mutualistic interactions between exotic ants and honeydew producers in the Yambaru district of Okinawa Island, Japan. Ecological Research, 26, 931–941. 10.1007/s11284-011-0851-2

[ece39073-bib-0064] Tanaka, H. O. , Haraguchi, T. F. , Tayasu, I. , & Hyodo, F. (2019). Stable and radio‐isotopic signatures reveal how the feeding habits of ants respond to natural secondary succession in a cool‐temperate forest. Insectes Sociaux, 66, 37–46. 10.1007/s00040-018-0665-0

[ece39073-bib-0065] Thibodeau, L. , Raymond, P. , Camire, C. , & Munson, A. D. (2000). Impact of precommercial thinning in balsam fir stands on soil nitrogen dynamics, microbial biomass, decomposition, and foliar nutrition. Canadian Journal of Forest Research, 30, 229–238. 10.1139/x99-202

[ece39073-bib-0066] Thomas, M. L. , Becker, K. , Abbott, K. , & Feldhaar, H. (2010). Supercolony mosaics: two different invasions by the yellow crazy ant, *Anoplolepis gracilipes*, on Christmas Island, Indian Ocean. Biological Invasions, 12, 677–687. 10.1007/s10530-009-9473-9

[ece39073-bib-0067] Thomas, S. C. , Halpern, C. B. , Falk, D. A. , Liguori, D. A. , & Austin, K. A. (1999). Plant diversity in managed forests: Understory responses to thinning and fertilization. Ecological Applications, 9, 864–879. 10.1890/1051-0761(1999)009[0864:PDIMFU]2.0.CO;2

[ece39073-bib-0068] Torchin, M. E. , Lafferty, K. D. , Dobson, A. P. , McKenzie, V. J. , & Kuris, A. M. (2003). Introduced species and their missing parasites. Nature, 421, 628–630. 10.1038/nature01346 12571595

[ece39073-bib-0069] Tsuji, K. , & Tsuji, N. (1996). Evolution of life history strategies in ants: Variation in queen number and mode of colony founding. Oikos, 76, 83–92. 10.2307/3545750

[ece39073-bib-0070] Vitousek, P. M. , D'Antonio, C. M. , Loope, L. L. , & Westbrooks, R. (1996). Biological invasions as global environmental change. American Scientist, 84, 468–478.

[ece39073-bib-0071] Vonshak, M. , & Gordon, D. M. (2015). Intermediate disturbance promotes invasive ant abundance. Biological Conservation, 186, 359–367. 10.1016/j.biocon.2015.03.024

[ece39073-bib-0072] Wang, Q. , Jin, S. , & Ruan, X. (2009). Ecological explanations for successful invasion of exotic plants. Frontiers of Biology in China, 4, 271–281. 10.1007/s11515-009-0032-7

[ece39073-bib-0073] Weir, S. M. , & Salice, C. J. (2012). High tolerance to abiotic stressors and invasion success of the slow growing freshwater snail, *Melanoides tuberculatus* . Biological Invasions, 14, 385–394. 10.1007/s10530-011-0084-x

[ece39073-bib-0074] Weng, S. H. , Kuo, S. R. , Guan, B. T. , Chang, T. Y. , Hsu, H. W. , & Shen, C. W. (2007). Microclimatic responses to different thinning intensities in a Japanese cedar plantation of northern Taiwan. Forest Ecology and Management, 241, 91–100. 10.1016/j.foreco.2006.12.027

[ece39073-bib-0075] Wilcove, D. S. , Rothstein, D. , Dubow, J. , Phillips, A. , & Losos, E. (1998). Quantifying threats to imperiled species in the United States. BioScience, 48, 607–615. 10.2307/1313420

[ece39073-bib-0076] Yamauchi, K. , Furukawa, T. , Kinomura, K. , Takamine, H. , & Tsuji, K. (1991). Secondary polygyny by inbred wingless sexuals in the dolichoderine ant *Technomyrmex albipes* . Behavioral Ecology and Sociobiology, 29, 313–331. 10.1007/BF00165955

[ece39073-bib-0077] Yamauchi, K. , & Ogata, K. (1995). Social structure and reproductive systems of tramp versus endemic ants (Hymenoptera: Formicidae) of the Ryukyu Islands. Pacific Science, 49, 55–68.

