# Supplementary material for: Resilience of native ant community against invasion of exotic ants after anthropogenic disturbances of forest habitats
Source: Ecol Evol. 2022 Jul 11;12(7):e9073. doi: 10.1002/ece3.9073 (PMC9272207; doi:10.1002/ece3.9073)
Supplement: Supplementary file 11 — Supplementary Material [file ECE3-12-e9073-s008.docx]

**Supplementary Information for**

**Resilience of native ant community against invasion of exotic ants after anthropogenic disturbances of habitats in the forest**

Hiroyuki Shimoji^1,^*, Mayuko Suwabe^2,3,^*, Tomonori Kikuchi^4^, Hitoshi Ohnishi^5^, Hirotaka Tanaka^6^, Kengo Kawara^7^, Yusuke Hidaka^6^, Tsutomu Enoki^7^, Kazuki Tsuji^6,†^

1 Department of Bioscience, School of Science and Technology, Kwansei Gakuin University, Hyogo, 669-1337, Japan

2 Okinawa Environmental Research Support Section, Okinawa Institute of Science and Technology Graduate University, Okinawa 904-0495, Japan

3 Biodiversity and Biocomplexity Unit, Okinawa Institute of Science and Technology Graduate University, Okinawa 904-0495, Japan

4 Marine Biosystems Research Center, Chiba University, Chiba, 288-0014, Japan

5 Kanto Regional Environment Office, Ministry of the Environment Government of Japan, Saitama 330-9720, Japan

6 Faculty of Agriculture, University of the Ryukyus, Okinawa 903-0213, Japan

7 Faculty of Agriculture, Kyusyu University, Fukuoka 819-0395, Japan

* These authors contributed equally to this work.

^†^ Correspondence: Kazuki Tsuji, Faculty of Agriculture, University of the Ryukyus, Okinawa 903-0213, Japan.

E-mail: tsujik@u-ryukyu.ac.jp

Tel. & Fax: +81 98 895 8797


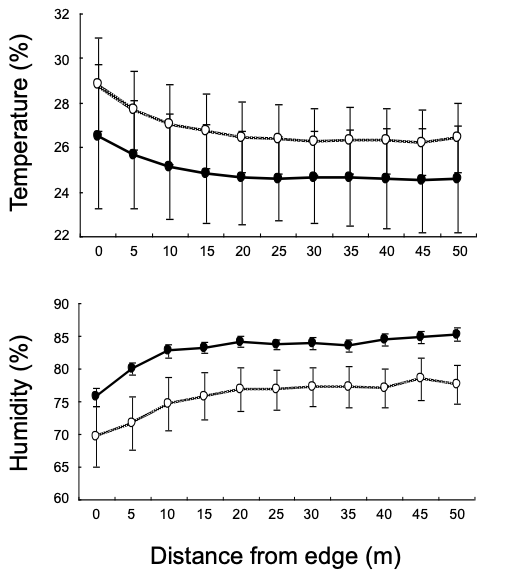


**Figure S1.** Relationships between temperature and humidity and distance from the road edge. Open circles and filled circles represent Iji (a roadside site of 5 years since road construction,) and Okuni (a roadside site of 25 years since road construction), respectively. Data were collected six times for each study point during June to November 2004 in the daytime (10 am -3 pm) on the same sunny or cloudy days. Values are means ± SD over 30 data points (6 measurements x 5 transects), each.
